# Quantifying interspecific variation in dispersal ability of noctuid moths using an advanced tethered flight technique

**DOI:** 10.1002/ece3.1861

**Published:** 2015-12-15

**Authors:** Hayley B. C. Jones, Ka S. Lim, James R. Bell, Jane K. Hill, Jason W. Chapman

**Affiliations:** ^1^ Department of AgroEcologyRothamsted ResearchHarpendenHertfordshireUK; ^2^ Department of BiologyUniversity of YorkYorkUK; ^3^ Environment and Sustainability InstituteUniversity of ExeterPenrynCornwallUK

**Keywords:** Flight behavior, flight capability, flight mill, Lepidoptera, migration, Noctuidae

## Abstract

Dispersal plays a crucial role in many aspects of species' life histories, yet is often difficult to measure directly. This is particularly true for many insects, especially nocturnal species (e.g. moths) that cannot be easily observed under natural field conditions. Consequently, over the past five decades, laboratory tethered flight techniques have been developed as a means of measuring insect flight duration and speed. However, these previous designs have tended to focus on single species (typically migrant pests), and here we describe an improved apparatus that allows the study of flight ability in a wide range of insect body sizes and types. Obtaining dispersal information from a range of species is crucial for understanding insect population dynamics and range shifts. Our new laboratory tethered flight apparatus automatically records flight duration, speed, and distance of individual insects. The rotational tethered flight mill has very low friction and the arm to which flying insects are attached is extremely lightweight while remaining rigid and strong, permitting both small and large insects to be studied. The apparatus is compact and thus allows many individuals to be studied simultaneously under controlled laboratory conditions. We demonstrate the performance of the apparatus by using the mills to assess the flight capability of 24 species of British noctuid moths, ranging in size from 12–27 mm forewing length (~40–660 mg body mass). We validate the new technique by comparing our tethered flight data with existing information on dispersal ability of noctuids from the published literature and expert opinion. Values for tethered flight variables were in agreement with existing knowledge of dispersal ability in these species, supporting the use of this method to quantify dispersal in insects. Importantly, this new technology opens up the potential to investigate genetic and environmental factors affecting insect dispersal among a wide range of species.

## Introduction

Dispersal is a key facet of species' ecology and evolution, and it has profound effects on population dynamics, gene flow, and range size (Clobert et al. 2001; Bowler and Benton 2005; Lester et al. 2007). Increasing our understanding of dispersal is of particular importance in an environment of accelerating climate change and habitat fragmentation (Hughes et al. 2007; Gibbs et al. 2009) because dispersal is important for range shifting (Pearson and Dawson 2003) and meta‐population dynamics (Hanski et al. 2000). However, obtaining direct measures of dispersal ability can be challenging, especially in insects, making it important to develop new tools for measuring species' flight capability.

Over the past 50 years, a variety of laboratory techniques has been developed to measure flight ability of insects under controlled and experimental conditions, including methods for measuring free‐flying insects (Kennedy and Booth 1963) as well as tethered individuals (Dingle 1965). Insects can be tethered in ways that allow them to change their orientation (in so‐called “flight simulators”, allowing identification of consistent seasonal migration directions (Mouritsen and Frost 2002; Nesbit et al. 2009). Other tethered‐flight techniques enable insects to repeatedly take‐off and land and thus allow assessment of flight propensity (Gatehouse and Hackett 1980), as well as assessment of migratory tendency through presence or absence of prolonged flight (Attisano et al. 2013). Insects can also be tethered on a flight mill that allows them to fly round in a circle to assess maximum flight duration and distance within a set period, e.g. over the course of a night for nocturnal migrants (Chambers et al. 1976; Beerwinkle et al. 1995; Zhang et al. 2009). Here, we extend these previous methods, and we describe and test a new tethered flight apparatus for quantifying flight ability in moths. This technique involves a roundabout‐style apparatus allowing flight distance, duration and speed to be quantified on the same individual insect. The key attributes of the apparatus are; compact multiple units allowing many individuals to be recorded simultaneously; very low friction bearings and magnetic suspension system to minimize the degree of friction associated with turning the arm during flight; and a lightweight but rigid tethered flight arm, allowing a wide range of species to be flown (from 10 mm to 40 mm forewing length, ~10–1000 mg mass). The system for attachment of the insect to the flight mill by a rigid wire handle attached to the top of the thorax allows for ease of handling, facilitating weighing and feeding and minimizing stress to the insect during preparation for flight. This system records flight distance to the nearest 10 cm every 5 sec, providing the most fine‐scale flight data currently available. We have produced bespoke software for downloading and summarizing flight data.

Here, we describe the apparatus and illustrate its capabilities by using it to examine differences in flight ability of 24 species of British noctuid moths. We have chosen this family to illustrate the potential of the apparatus because the family includes species with a wide range of different dispersal abilities, body sizes, and life histories. We describe the improved tethered flight mill system and the measures of flight that are recorded. We demonstrate that differences in flight mill performance reflect differences in dispersal abilities under natural conditions, and discuss how the apparatus could be used to better understand dispersal ability in insects in the future.

## Methods

### Tethered flight mills and their operation

**Figure 1:**
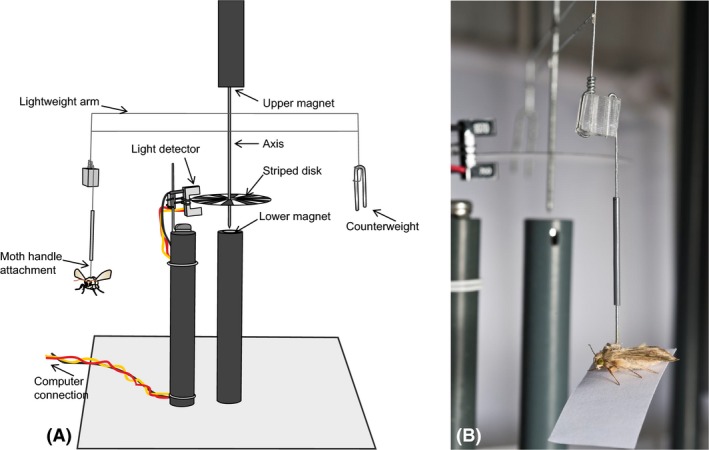
Tethered flight mill. (A) Labeled diagram of an individual flight mill. (B) Close up of the method of attaching the moth to the flight mill. Flight mills are patented (PCT/GB2014/052466). Moth shown is *Helicoverpa armigera* (species mean weight 0.200 g, wing length 15–20 mm).

An illustration of a flight mill is shown in Figure 1 (Patent: Lim et al. 2013). Each mill consists of a lightweight arm suspended between two magnets. This magnet suspension provides an axis with very little resistance, so even relatively weak fliers can turn the mill successfully. The novel aspect of our design that permits interspecific comparison of flight is the mill arm, which is very lightweight but suitably rigid due to a unique construction method (Patent: Lim et al. 2013). The insect is attached to one end of the arm as shown in Figure 1B and flies in a circular trajectory with a circumference of 50 cm. A disk with a banded pattern is attached to the axis so that it turns with the arm, and a light detector detects the movement of the bands to record the distance flown and the flight speed. The tethered flight mill system currently has 16 channels (arms) allowing 16 individual insects to be flown simultaneously (but can be extended to include more channels). Flight data are automatically downloaded to a computer. The embedded microcontroller board records the distance flown by the insect to the nearest 10 cm and updates the computer with the distance travelled in five second intervals. An example of the data generated by the flight mill can be found in Appendix S1.

**Figure 2:**
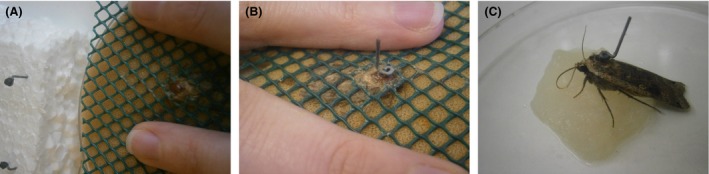
Preparing moths for tethered flight. (A) Removal of scales from thorax. (B) Attachment of flight handle with contact adhesive. (C) Feeding with honey solution.

To prevent damage to moths when preparing them for the flight mills, individuals were kept inactive in a domestic fridge and then restrained under netting to fix the attachment (Fig. 2). Scales were removed from the upper surface of the thorax using sticky tape, and then “handles” attached with contact adhesive. This system of having a short handle attached to the moth facilitates weighing and feeding prior to the insect being attached to the flight mills. Data recorded by the flight mills are measures of distance flown (m), time spent flying (s), and flight speed (m/s) (Table 1). These data can be used to analyse measures of distance, duration and speed of specific flights (e.g. the first flight of the night, or the longest flight), and derive additional variables. Flight data for each individual moth are processed using a script written in Matlab (The MathWorks Inc. 2012) to extract the beginning and end time of each individual flight and calculate each flight's duration, distance, and average speed. Because flight duration is always rounded up to the nearest 5 sec by the recording equipment, any small movement by an insect on the mill is recorded as a flight of 5 sec, therefore in our validation of the apparatus, we only analysed data for flights of 10 sec or longer. The maximum speed (calculated from the greatest distance travelled in any 5 sec interval) is also extracted. These flight data are processed in R (R Core Team 2013) to extract a total of 16 tethered flight variables (listed in Table 1).

**Table 1:** Measured and derived tethered flight performance variables extracted from flight mill data. Raw data are distance, duration, average speed, and maximum speed of individual flights ≥10 sec

Tethered flight variable	Definition	Units	PCA label
Total distance	Sum of distance covered by all flights	Metres	Distance 1
Total duration	Sum of duration of all flights	Seconds	Duration 1
Number of flights	Count of flights	Numeric	NumFlights
Average flight distance	Mean of distances of flights	Metres	Distance 2
Average flight duration	Mean of duration of flights	Seconds	Duration 2
Average flight speed	Mean of the speeds of individual flights (calculated as distance/duration)	Metres/sec	Speed 1
Maximum speed attained	Greatest distance attained in any 5 sec interval/5 – of the whole night	Metres/sec	Speed 2
First flight distance	Distance of first flight of the night	Metres	Distance 3
First flight duration	Duration of first flight of the night	Seconds	Duration 3
First flight average speed	Speed of first flight of the night (calculated as distance/duration)	Metres/sec	Speed 3
First flight max speed	Greatest speed attained in any 5 sec interval of the first valid flight	Metres/sec	Speed 4
Furthest flight distance	Distance travelled in the flight of greatest distance of the whole night	Metres	Distance 4
Longest flight distance	Distance travelled in the flight of greatest duration of the whole night	Metres	Distance 5
Longest flight duration	Duration of the flight with greatest duration	Seconds	Duration 4
Longest flight average speed	Speed of the flight with greatest duration (calculated as distance/duration)	Metres/sec	Speed 5
Longest flight max speed	Greatest speed attained in any 5 sec interval of the flight of greatest duration	Metres/sec	Speed 6

### Validating flight mill data

**Figure 3:**
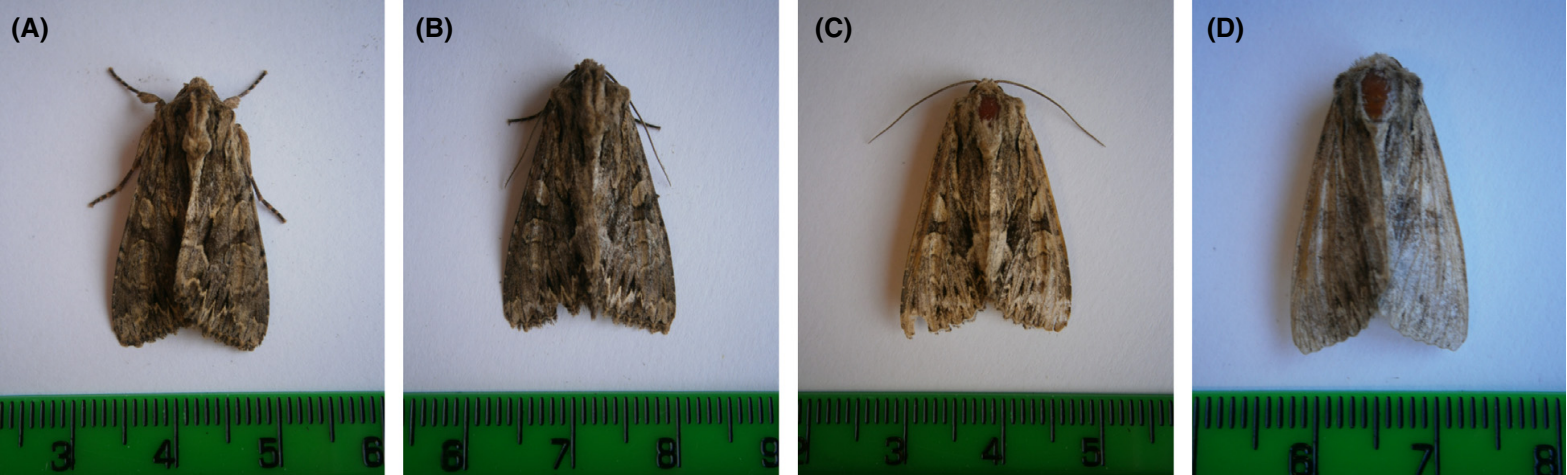
Wing wear categories as per Thomas (1983) demonstrated in A*pamea monoglypha*. (A) Fresh (4). (B) Good (3). (C) Poor (2). (D) Worn (1).

Noctuid moths captured in light‐traps on site at Rothamsted Research, Harpenden, UK (51.809°N, −0.356°W) during summer 2013 were used in flight mill trials. Visual inspection of wing wear was used to ensure only recently emerged adults were flown, to constrain any variation in flight according to adult age. Following Thomas (1983), wing wear was assessed on a four point scale; fresh (4), good (3), poor (2), and worn (1), and only category 3 and 4 insects were used (Fig. 3A and B).

Insects were kept in a domestic fridge and flown the following night after being caught. About two hours prior to flight, moths were removed from the fridge, weighed, and then given 20% honey solution *ad libitum*. They were then reweighed to verify feeding and attached to the flight mill with a piece of paper to hold on to and left until the lights were switched off at 21:00 BST. Each moth was flown on only one night. The flight mills were housed in a controlled environment insectary room at 18°C and 18L: 6D, which is equivalent to midsummer in the UK. Lights gradually changed during the hour before and after the night‐time dark period to simulate dawn and dusk. Multivariate analyses were carried out to examine which of the 16 tethered flight variables recorded by the apparatus (Table 1) were the most biologically informative.

In order to test the assumption that tethered flight performance reflects natural dispersal behavior in the wild, all study species were assigned to a mobility category based on two sources of information. First, we examined Rothamsted Insect Survey suction trap (Macaulay et al. 1988) data on the occurrence of moths in traps 12.2 m above the ground over the period 2000–2009 (Wood et al. 2009). We used the presence of a study species in the top 25% of all species caught 12.2 m above the ground to infer a strong likelihood of the study species to engage in long distance dispersal (Wood et al. 2009).

Secondly, we carried out a survey asking experts to classify the study species according to whether species were relatively sedentary, mobile or very mobile. The experience and opinion of lepidopterists has been shown to be a valid tool in quantifying dispersal ability (Stevens et al. 2010; Burke et al. 2011). Five experts designated each of the 24 study species into one of three dispersal categories, based on the experts' opinion and knowledge of the species' relative dispersal ability. The categories were sedentary (0), mobile (1), and very mobile (2; Table 2).

**Table 2:** Responses to expert survey on noctuid moth mobility. Five experts categorized species as relatively sedentary, mobile, or very mobile which corresponds to 0, 1 or 2 mobility points in the table below

Species	Expert 1	Expert 2	Expert 3	Expert 4	Expert 5	Mean points
*Agrotis exclamationis*	2	1	1	1	0	1
*Agrotis puta*	2	1	1	1	1	1.2
*Amphipoea oculea*	1	1	0	1	1	0.8
*Amphipyra pyramidea*	1	1	0	1	1	0.8
*Apamea monoglypha*	2	2	1	1	1	1.4
*Autographa gamma*	2	2	2	2	2	2
*Axylia putris*	1	1	0	1	0	0.6
*Hoplodrina alsines*	2	1	0	1	0	0.8
*Hoplodrina ambigua*	2	1	1	1	2	1.4
*Hydraecia micacea*	1	1	0	1	0	0.6
*Lacanobia oleracea*	1	1	0	1	0	0.6
*Mesapamea secalis*	2	1	0	1	0	0.8
*Mesapamea didyma*	2	1	0	1	0	0.8
*Mythimna impura*	2	1	0	1	0	0.8
*Mythimna pallens*	2	1	0	1	0	0.8
*Noctua comes*	2	2	1	1	0	1.2
*Noctua janthe*	2	2	1	1	1	1.4
*Noctua pronuba*	2	2	2	2	2	2
*Ochropleura plecta*	1	1	1	1	1	1
*Omphaloscelis lunosa*	2	1	0	1	1	1
*Phlogophora meticulosa*	2	2	0	2	2	1.6
*Xestia c‐nigrum*	2	1	1	1	2	1.4
*Xestia triangulum*	1	1	1	1	0	0.8
*Xestia xanthographa*	2	1	0	1	0	0.8

We combined the two sources of information on dispersal ability to place the 24 study species into three categories: “low”, “medium”, and “high” mobility (Table 3). An ANOVA was used to compare tethered flight variables among moth species assigned to these three mobility categories.

**Table 3:** Summary table of individual moth species flown on tethered flight mills. All individuals were males

Species	*N* flown	Suction trap score	Expert opinion	Score	Mobility category	Total distance (m)	Maximum speed (m/sec)
*Agrotis exclamationis*	18	1	1	2.0	Medium	6935	1.458
*Agrotis puta*	8	1	1.2	2.2	Medium	597	0.743
*Amphipoea oculea*	11		0.8	0.8	Low	1580	0.962
*Amphipyra pyramidea*	14		0.8	0.8	Low	12352	1.799
*Apamea monoglypha*	39	1	1.4	2.4	High	9036	2.059
*Autographa gamma*	13	1	2	3.0	High	5168	1.535
*Axylia putris*	14		0.6	0.6	Low	2474	0.979
*Hoplodrina alsines*	13		0.8	0.8	Low	2647	1.152
*Hoplodrina ambigua*	13		1.4	1.4	Medium	1166	0.974
*Hydraecia micacea*	23		0.6	0.6	Low	2647	1.163
*Lacanobia oleracea*	16		0.6	0.6	Low	3756	1.352
*Mesapamea didyma*	10	1	0.8	1.8	Medium	3598	1.112
*Mesapamea secalis*	16	1	0.8	1.8	Medium	3574	1.046
*Mythimna impura*	11		0.8	0.8	Low	1581	0.807
*Mythimna pallens*	19		0.8	0.8	Low	2675	0.882
*Noctua comes*	26		1.2	1.2	Medium	6548	1.474
*Noctua janthe*	13		1.4	1.4	Medium	4489	1.215
*Noctua pronuba*	37	1	2	3.0	High	11596	1.623
*Ochropleura plecta*	20		1	1.0	Low	626	0.697
*Omphaloscelis lunosa*	16		1	1.0	Low	1693	1.286
*Phlogophora meticulosa*	10	1	1.6	2.6	High	9501	1.877
*Xestia c‐nigrum*	59	1	1.4	2.4	High	5903	1.17
*Xestia triangulum*	12		0.8	0.8	Low	5254	1.478
*Xestia xanthographa*	25	1	0.8	1.8	Medium	4193	0.936

Mobility category was assigned by summing scores from suction trap data and expert survey. One point was assigned if species were in the top 25% of species caught in Rothamsted Insect Survey (RIS) suction traps (mean yearly catch over period 2000–2009). Expert opinion was the mean value of responses where five experts were asked to assign species to categories of low (0), medium (1), and high (2) mobility (see Table 2). “Score” sums these two methods of classification and mobility category was assigned according to thresholds: ≤1 = Low, >1 to ≤2 = Medium and >2 = High. Species mean values for the tethered flight variables “Total distance flown overnight” and “maximum speed” are also shown.

## Results

### Characterizing dispersal ability with tethered flight

**Figure 4:**
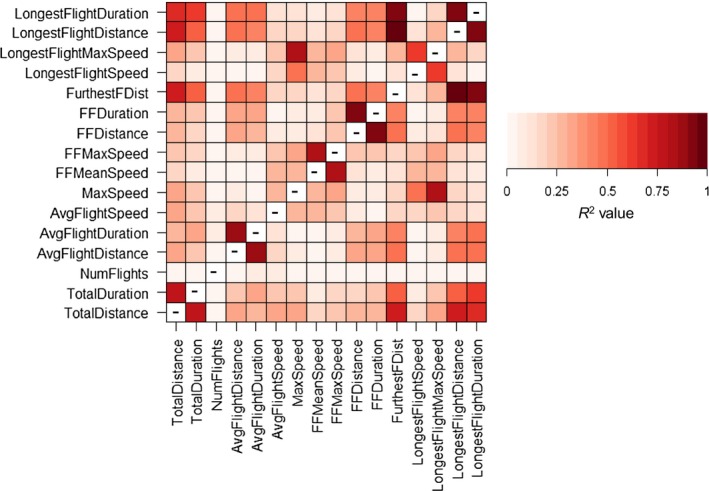
Matrix of pair‐wise correlations of the sixteen tethered flight variables outlined in Table 1. A dash indicates a cell where a correlation value has not been computed.

**Figure 5:**
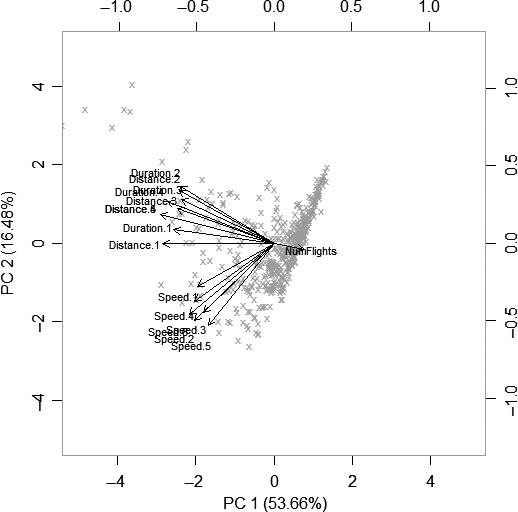
Principal components analysis biplot of the 16 tethered flight mill variables listed in Table 1. The two first principal components are plotted with the proportion of variance explained by each component printed next to the axes label which together explain >70% of variation in the data. Crosses indicate the 456 male individuals in the data set; the top and right axes show principal component scores of the individuals. The arrows indicate the principal component loadings of the different tethered flight variables.

Significantly more males were caught than females in light traps, and so our sample sizes for flight mill validation were higher for males (495 individuals) than females (122 individuals). Given that there is likely to be intraspecific variation in flight between males and females (Berwaerts et al. 2006), and in order to maximize the number of species we studied, all flight trials were based only on males. In order to obtain robust measures for species, and to account for intra‐specific variation in flight, we only included species with ≥8 individuals flown (hence we measured 456 individuals in total, median = 15 individuals per species, from 24 species; Table 3). Many of the 16 tethered flight variables were highly correlated (Fig. 4) and a Principal Components Analysis confirmed redundancy in measures (Fig. 5), but that measurement of flight distance/duration and flight speed characterized different aspects of dispersal. A Canonical Variates Analysis (Table 4) indicated that measures of flight speed best distinguished among moth study species. Thus we concluded that that “total distance flown overnight” and “maximum speed” were the best tethered flight variables to analyse that captured most of the variation in flight in our study species.

**Table 4:** Canonical Variates Analysis was performed on the 16 tethered flight variables (outlined in Table 1)

Tethered flight measurement	CV1 (45.46)	CV2 (14.75)	CV3 (10.57)	CV4 (7.26)	CV5 (7.1)
AvgFlightDistance	−0.0002	0.0001	0.0004	0	0.0001
AvgFlightDuration	0.0001	−0.0002	−0.0004	0	−0.0002
AvgFlightSpeed	0.8207	−3.5807	−0.5541	−2.5477	2.1785
FFDistance	0.0001	0.0003	−0.0002	−0.0004	0.0002
FFDuration	−0.0001	−0.0002	0.0002	0.0002	−0.0001
FFMaxSpeed	0.3871	1.3461	−1.1428	0.7091	−0.8464
FFMeanSpeed	−1.0561	−1.3326	0.902	0.8578	3.5797
FurthestFDist	−0.0001	−0.0001	−0.0001	0.0005	0
LongestFlightDistance	−0.0001	−0.0001	−0.0001	0.0005	0
LongestFlightDuration	0.0001	0.0002	0	−0.0007	0.0001
LongestFlightMaxSpeed	1.1193	0.4216	1.392	−1.12	1.3157
LongestFlightSpeed	0.0129	0.9717	0.4125	−1.8829	0.8313
MaxSpeed	1.302	0.5732	−1.5167	1.1944	−1.9183
NumFlights	0.0095	−0.0076	0.0325	−0.0066	0.0087
TotalDistance	0.0001	0.0001	0.0001	−0.0004	−0.0003
TotalDuration	0	−0.0002	0.0001	0.0003	0.0002

Loadings values of the variables in the first five canonical variates are shown. Values in brackets next to CV number are the percentage variance in the dataset accounted for by that canonical variate.

### Validating flight mill data

**Figure 6:**
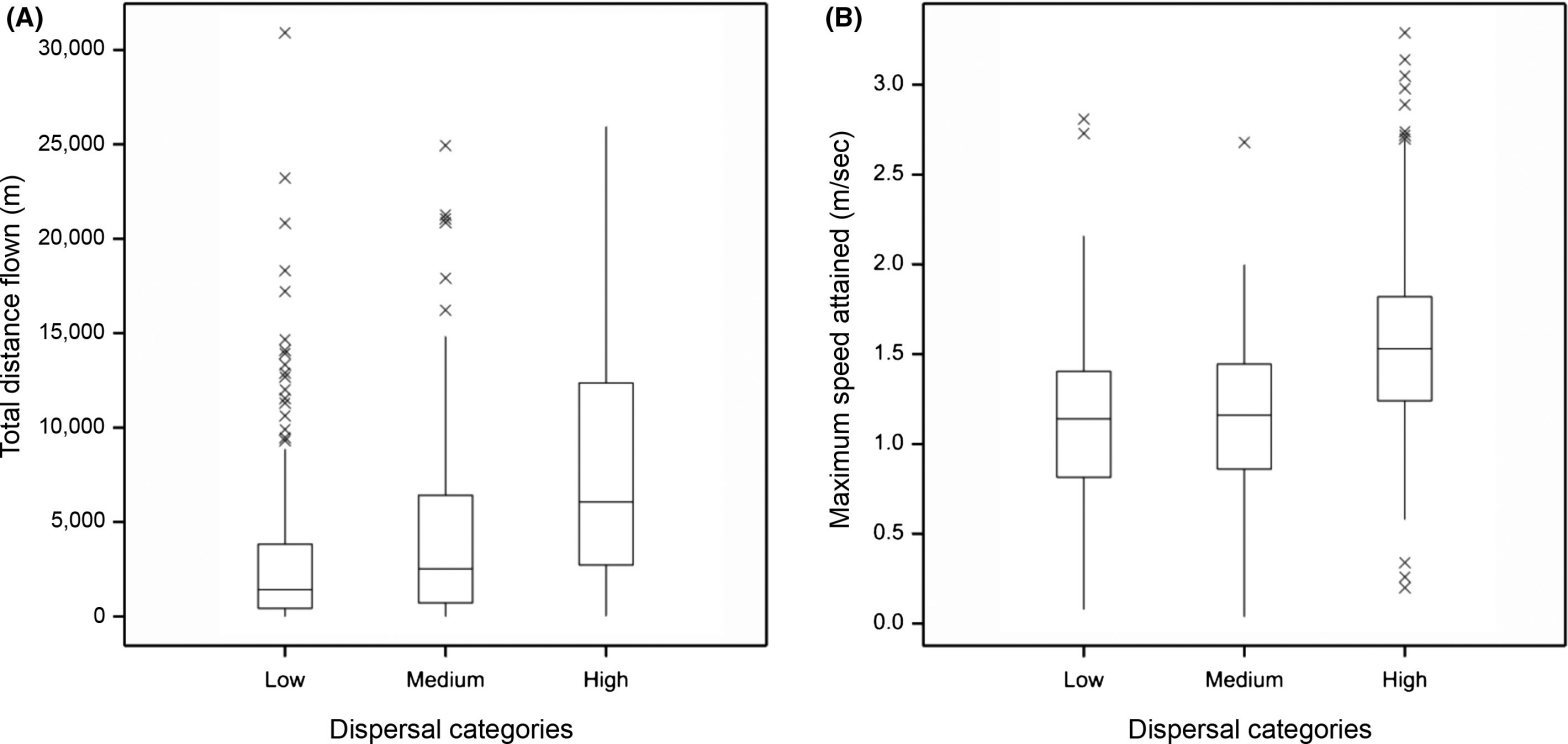
Boxplots showing (A) total distance flown and (B) maximum speed attained on tethered flight mills of 456 individuals assigned to three dispersal categories according to their species (Table 3). Boxes span the interquartile range of values, with the line dissecting the box indicating the median. Whiskers extend to 1.5 times the interquartile range beyond the quartiles. Beyond this outliers are plotted as a cross.

Individuals from the 24 study species were assigned to mobility categories (low, medium or high) according to their species scores in Table 3. Mobility category had a significant effect on both flight distance and speed (total distance flown: *F*_2,21_ = 8.69, *P* = 0.002; maximum speed: *F*_2,21_ = 4.61, *P* = 0.022; Fig. 6). A Tukey post‐hoc test confirmed that the medium and low mobility groups had significantly shorter flight distances than the high group, and the low mobility group had slower flight speeds than the high group. Information on total distance and maximum speed of the study species are plotted as boxplots (Fig. 6).

## Discussion

In this study, we describe and test a new tethered flight system that has enabled us to fly a wide variety of noctuid moth species in controlled laboratory conditions (Patent: Lim et al. 2013). While other studies have used tethered flight mills to examine intra‐specific variation in flight performance, e.g. in relation to sex, population, age and levels of sexual maturity within species (Mcanelly 1986; Schumacher et al. 1997; Berwaerts et al. 2006; Taylor et al. 2010), this is the first study that has compared flight performances across a number of species spanning a range of sizes. Our novel flight mill design with magnetic suspension of the axis and a unique lightweight but rigid arm is sufficiently lightweight and low friction for the smallest species but strong enough for the largest species, and so facilitates the study of a wide range of species. Our apparatus currently allows the testing of 16 individuals at the same time, and the software extracts 16 flight variables for subsequent analysis. This enables the user to look at many aspects of the speed, distance and duration of flights and pattern of flight performance overnight. For our study species, measures of “total distance flown overnight” and “maximum speed” were the most informative for distinguishing among the 24 study species, but the range of flight variables recorded provides substantial flexibility in the types of experimental studies that could be carried out.

### Flight mill validation

We showed that the tethered flight data obtained on the apparatus are representative of natural flight ability of species, supporting the usefulness of the apparatus in investigations of insect dispersal. Species placed in the high mobility category (such as *Noctua pronuba* and *Autographa gamma;* group mean flight distance = 8178 m*)* had mean flight distances 2.5 times that of species in the low mobility category (such as *Axylia putris* and *Hydraecia micaea*; group mean = 3263 m). Four of the five species in the “high” mobility group (*A. gamma*,* N. pronuba, Phlogophora meticulosa* and *Xestia c‐nigrum*) are migrants (Waring et al. 2009; Chapman et al. 2010), whereas there is very little published information on dispersal ability in other species. This lack of dispersal information was reflected in the expert survey information (Table 2) where there was some lack of consensus on which moths belonged in the “low” and “medium” categories. This lack of consensus may explain why our analyses were generally less capable of distinguishing between the low and medium groups of species, compared with the high group. All the study species are noctuids and are relatively mobile compared with some other macro‐moth families (e.g. Geometridae), but nonetheless there is variation in dispersal ability among these species which was evident in flight mill data. We therefore conclude that the tethered flight mills are an important new tool to elucidate dispersal ability in a wider range of species than has been possible previously.

### Limitations of the flight mill system

Our tethered flight mill system has some limitations as a tool to assess dispersal, which are common to most tethered flight techniques. The tether restricts natural flight somewhat as it may obstruct wing‐flapping, especially in species which employ a “clap‐and‐fling” style of flight, e.g. butterflies (Srygley and Thomas 2002). Our preliminary flight observations concluded that geometrid moths' wing‐flapping was obstructed by the tether, but noctuid moths did not appear to be hindered. Flying on a tether also means that the insects do not have to produce sufficient lift to overcome their body weight and thus are not expending as much energy as free flying insects (Riley et al. 1997).

It is more complex to interpret how distances flown on the flight mill might relate to dispersal distances in the wild. It is difficult to simulate all the cues that an insect may require to fly, which is especially important if flight propensity is of interest (Colvin and Gatehouse 1993), and so insects may not behave naturally when tethered. For example, moths may not receive appropriate cues to take off, or once in flight, the absence of appropriate cues may prolong the insect's flight and delay landing. In addition, the lack of tarsal contact with the ground and the inability to land will likely encourage insects to fly for greatly extended periods compared to natural flight (Gatehouse and Hackett 1980), and thus the flight mill measures are more likely to be representative of upper flight limits than normal flight activity. Conversely, the added physical effort of pushing the flight mill while flying may cause the insect to tire and cease flight more quickly than in the wild; however, this may be countered by the lower energy expenditure resulting from the lift provided by the tether.

Despite these criticisms, tethered flight mills are an invaluable tool in studying the flight performance of nocturnal and/or high flying insects for which no observation of natural flight duration and movement pathways may be possible. Tethered flight mills are valuable tools to demonstrate differences in dispersal ability among different groups, as evidenced by this study and others (Blackmer et al. 2004; Taylor et al. 2010).

### Potential for using flight mill system in new investigations

The tethered flight mills provide a platform to explore the relationship between measures of dispersal ability (such as flight speed and duration), and physiological, genetic and environmental factors that promote or inhibit flight. Insects can be flown after being caught from the wild, enabling assessment of the amount of variation in dispersal ability present in wild populations. Insects can also be flown having been reared under controlled conditions, which enables the effects of food availability, climate and disease levels during development on dispersal propensity to be assessed. The “handle” by which the moths are attached to the mill is small and light compared to many other set‐ups, enabling moths to be flown on sequential nights, and therefore age‐related changes in flight behavior can be quantified. Genetic and epigenetic factors affecting dispersal ability can also be assessed and compared across species.

In addition to the flight mill apparatus outlined in this paper, we also have flight mills with longer arm lengths that we have used to fly large, powerfully flying species such as the European hornet (*Vespa crabro)*, hawk moths (Sphingidae), bumblebees (*Bombus terrestris*), and honeybees (*Apis mellifera*); and flight mills with extremely small and lightweight arms that have been used to quantify the flight ability of small, weak‐flying insects including brown planthoppers (*Nilaparvata lugens)* and mosquitoes (*Aedes aegypti*), weighing <1 mg. We are currently developing calibration methods that will enable the comparison of distances flown on different arm types, thereby opening up new possibilities to compare a much wider range of taxa. We conclude that our new tethered flight apparatus provides a robust technique to assess the flight ability of insects. This new technique opens up the potential to quantify the dispersal abilities of a much wider range of species for which current knowledge of dispersal is lacking, and to address a plethora of scientific questions about factors affecting insect dispersal.

## Data Accessibility

Data will be uploaded to http://datadryad.org/.

The flight mills are subject to a patent (Patent number: PCT/GB2014/052466), but collaboration is welcomed or we recommend you contact the Knowledge Exchange and Commercialization team at Rothamsted (andrew.spencer@rothamsted.ac.uk).

## Conflict of Interest

None declared.

## Supporting information


**Appendix S1.** Example of raw data generated by the tethered flight mill for 8 moths in one night.
